# *Notes from the Field:* HIV Infection Investigation in a Rural Area — West Virginia, 2017

**DOI:** 10.15585/mmwr.mm6708a6

**Published:** 2018-03-02

**Authors:** Mary E. Evans, Sarah M. Labuda, Vicki Hogan, Christine Agnew-Brune, John Armstrong, Amarnath Babu Periasamy Karuppiah, Deborah Blankinship, Kate Buchacz, Kenya Burton, Sharon Cibrik, William Hoffman, Nathan Kirk, Chang Lee, Dondeena McGraw, M. Cheryl Bañez Ocfemia, Nivedha Panneer, Pamela Reynolds, Bridget Rose, Melinda Salmon, Melissa Scott, Antoine Thompson, David Wills, Sherri A. Young, Rahul Gupta, Loretta Haddy, Paul J. Weidle, Miguella Mark-Carew

**Affiliations:** ^1^Epidemic Intelligence Service, CDC; ^2^Division of HIV/AIDS Prevention, National Center for HIV, Viral Hepatitis, STD, and TB Prevention, CDC; ^3^Arkansas Department of Health; ^4^West Virginia Department of Health and Human Resources, Bureau for Public Health; ^5^Public Health Informatics Fellowship Program, CDC; ^6^Division of Global Health Protection, Center for Global Health, CDC; ^7^Division of STD Prevention, National Center for HIV, Viral Hepatitis, STD, and TB Prevention, CDC.

From January to July 2017, the West Virginia Department of Health and Human Resources (WV DHHR) identified 10 cases of human immunodeficiency virus (HIV) infection in three counties where HIV diagnoses typically range from six to 13 annually (*1*). In these counties, the spread of bloodborne pathogens via injection drug use (IDU) is a major public health concern, and risk reduction programs offering syringe services were not available, although they were available in other counties (*2*,*3*). As of July 2017, nine of the 10 persons identified were men who have sex with men (MSM), two of whom had reported a prior history of IDU. Coinfections with syphilis (five patients), hepatitis B virus (three), and hepatitis C virus (HCV) (two) were also documented. By September 2017, the sexual or injection contacts named by persons in the investigation expanded the original assessment area to encompass 15 counties, 14 of which were among the nation’s top 220 counties thought to be particularly vulnerable to rapid spread of HIV and HCV infections via IDU (*4*). The investigated counties share some characteristics with rural Scott County, Indiana, where an HIV outbreak was linked to IDU in 2015 (*5*), including a high prevalence of drug overdose deaths, prescription opioid sales, and unemployment.

WV DHHR and CDC reviewed HIV surveillance and partner services data to identify persons with HIV infection diagnosed in 2017 who resided in one of the 15 counties at the time of diagnosis. These included HIV-infected persons who were epidemiologically linked (sex or IDU partner) or molecularly linked (by closely related HIV nucleotide sequences at a distance of ≤0.005 substitutions per site) to at least one case diagnosed in 2017 who resided in one of the 15 counties at the time of diagnosis. In addition, information on available health care services was obtained through individual interviews with 18 local providers and five persons from the investigation who had HIV infection.

As of October 26, 2017, the investigation had identified 57 persons with diagnosed HIV infection, including 40 cases (73%) diagnosed in 2017 (Figure) and 17 cases diagnosed before 2017 that were epidemiologically linked (11 cases) or molecularly linked (six). Males accounted for 51 (89%) persons with HIV infection; 43 (75%) were white, and 28 (49%) were aged <30 years. The mode of transmission was male-to-male sexual contact in 34 cases (60%), IDU in five (9%), both male-to-male sexual contact and IDU in three (5%), heterosexual contact in two (4%), and unknown in 13 (23%). Ten (18%) persons had HIV Stage 3 (acquired immunodeficiency syndrome [AIDS]) at the time of diagnosis. All 40 persons with HIV infection diagnosed in 2017 had been linked to HIV care; 13 (77%) of the 17 persons diagnosed before 2017 had an HIV medical care visit in the past 6 months.

**FIGURE:**
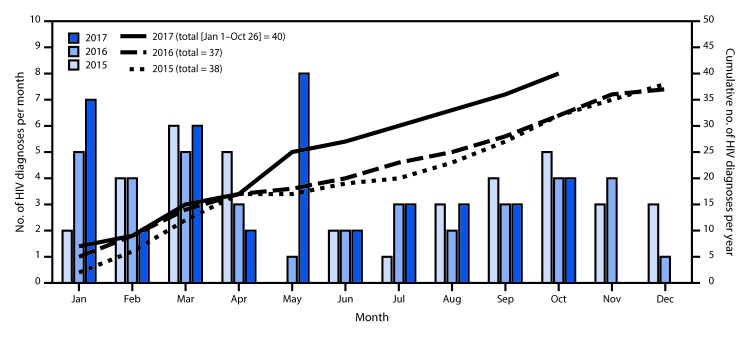
Number of HIV diagnoses per month and cumulative number of diagnoses per year — 15 West Virginia counties, 2015–2017 **Abbreviation:** HIV = human immunodeficiency virus.

Risk reduction programs that provide syringe services are available in three of the 15 counties (none of the original three counties in the investigation). HIV testing is available at all 15 county health department clinics. HIV medical care providers are available in three counties (including one of the original counties in the investigation). Ryan White HIV/AIDS Program case management services, which support organizations to deliver HIV care and treatment for low-income persons living with HIV infection, are available throughout the state. Providers who offer preexposure prophylaxis for prevention of HIV infection are available in clinic settings in four counties (including two of the original counties in the investigation). Local providers and persons with HIV infection who were interviewed described stigma, transportation from remote areas, and poor health literacy as challenges to HIV testing in rural areas.

WV DHHR implemented strategies to limit further transmission of HIV among the MSM population and among persons who inject drugs. WV DHHR is expanding access to HIV testing and plans to work with county health departments to implement additional preexposure prophylaxis clinics. For example, a geotargeted advertisement in the 15 counties on popular MSM-focused social media sites was purchased to encourage HIV testing. In addition, in November, WV DHHR selected 11 health care entities across West Virginia for funding, including entitites in five of the counties in this investigation, to support comprehensive community-level prevention programs that include syringe services programs where they are permitted and desired. Continued efforts are underway to further characterize the HIV transmission network and potential HIV transmission through IDU risk behavior among patients and their extended contact networks.
